# The Evolutionary Rates of HCV Estimated with Subtype 1a and 1b Sequences over the ORF Length and in Different Genomic Regions

**DOI:** 10.1371/journal.pone.0064698

**Published:** 2013-06-06

**Authors:** Manqiong Yuan, Teng Lu, Chunhua Li, Ling Lu

**Affiliations:** 1 Department of Pathology and Laboratory Medicine, Center for Viral Oncology, University of Kansas Medical Center, Kansas City, Kansas, United States of America; 2 University of Southern California, Los Angeles, California, United States of America; Saint Louis University, United States of America

## Abstract

**Background:**

Considerable progress has been made in the HCV evolutionary analysis, since the software BEAST was released. However, prior information, especially the prior evolutionary rate, which plays a critical role in BEAST analysis, is always difficult to ascertain due to various uncertainties. Providing a proper prior HCV evolutionary rate is thus of great importance.

**Methods/Results:**

176 full-length sequences of HCV subtype 1a and 144 of 1b were assembled by taking into consideration the balance of the sampling dates and the even dispersion in phylogenetic trees. According to the HCV genomic organization and biological functions, each dataset was partitioned into nine genomic regions and two routinely amplified regions. A uniform prior rate was applied to the BEAST analysis for each region and also the entire ORF. All the obtained posterior rates for 1a are of a magnitude of 10^−3^ substitutions/site/year and in a bell-shaped distribution. Significantly lower rates were estimated for 1b and some of the rate distribution curves resulted in a one-sided truncation, particularly under the exponential model. This indicates that some of the rates for subtype 1b are less accurate, so they were adjusted by including more sequences to improve the temporal structure.

**Conclusion:**

Among the various HCV subtypes and genomic regions, the evolutionary patterns are dissimilar. Therefore, an applied estimation of the HCV epidemic history requires the proper selection of the rate priors, which should match the actual dataset so that they can fit for the subtype, the genomic region and even the length. By referencing the findings here, future evolutionary analysis of the HCV subtype 1a and 1b datasets may become more accurate and hence prove useful for tracing their patterns.

## Introduction

The evolutionary analysis of hepatitis C virus (HCV) genetic sequences has entered a new era since the BEAST software (Bayesian Evolutionary Analysis by Sampling Trees) was released in 2003 [1]–[3]. Using this software, the retrospective changes in the HCV-infected population size can be illustrated as a Bayesian Skyline Plot (BSP) [4], [5], in which the population size is measured on the vertical axis, while the elapsed time is scaled on the horizontal axis [6]–[10]. In addition, a time-scale phylogenetic tree is able to represent the diverse histories, from the most recent common ancestor (tMRCA) to the most recent descendants [6]. Moreover, a phylogeographic tree can be reconstructed if the information on the sampling geographic locations is provided, which displays the transmission and migration histories of the virus [8], [11]. Perhaps the most important feature of the BEAST program is the implementation of a Bayesian statistical framework, which provides a role for prior knowledge or information [2]. Prior information, specifically a prior probability distribution, expresses one's uncertainty for a given parameter before the data is analyzed. It plays a critical role in generating the posterior, a distribution that combines the prior and the data information. If the sequences are sampled over a very short period of time, or at the extreme, a single time point, the evolutionary rate is completely determined by the prior information provided. In this sort of case, providing improper prior information might actually be worse than providing no information at all.

However, due to the shortage of historically archived samples, the estimation of an accurate prior HCV evolutionary rate has been difficult. Currently, the most widely used HCV rates are 7.9×10^−4^ substitutions/site/year for the E1 region and 5.0×10^−4^ for NS5B. Pybus et al. first used these rates to open the door to investigating the epidemic history of HCV [9], [12]–[14]. These rates have been applied as prior rates, for example, in the analysis of genotype 2 in West Africa [15], genotype 4 in Central Africa [16] and subtype 1b in China [7]. Collectively, these two rates have played an irreplaceable role in the study of HCV evolution in a variety of epidemic scenarios and for different genotypes and subtypes, and have therefore enabled very important information to be generated and invaluable inferences to be drawn.

Better HCV evolutionary rates may nevertheless still be estimated. It is known that the above mentioned NS5B rate was estimated based on 100 HCV subtype 1b sequences, each having a length of 222 nucleotides (nt) [12]. Ideally, they represent a single-lineage-among-host sequence dataset, i.e. they were determined from a cohort of women infected by a contaminated blood product (anti-D immunoglobulin) that had been generated from a single HCV-infected blood donation [17]. A recent study reported that a lower HCV evolutionary rate was estimated from such a dataset representing within-host isolates than a dataset representing multiple-lineage among-host isolates [18]. It is implied that the use of a single-lineage rate in a multiple-lineage setting may therefore underestimate the speed of viral evolution and thus suggest an earlier timing for the estimated HCV population growth and the correlated historical events.

HCV is known to have six genotypes and many subtypes. They have been shown to have different geographic distribution patterns reflecting the different selective pressures that they have undergone [1]. Therefore, they are supposed to have different evolutionary histories and substitution rates. The latter was verified recently. Gray et al. reported that the genome-wide evolutionary rate for subtype 1a is approximately 19% higher than that for subtype 1b [18]. Because among genotypes higher genetic distances are seen, their differences in evolutionary rates can be larger. Even for a single HCV strain in the same genomic region, the rates may be dissimilar over different lengths. Therefore, it is believed that if the selected prior rates can best fit for the genotypes, subtypes, genomic regions and even the lengths of a given dataset, the posterior inference would be more accurate.

Currently, HCV detection and genotyping is primarily based on the determination of partial sequences in two regions, a 576 nt Core-E1 and a 324 nt NS5B region, corresponding to the nucleotide numbering 738–1313 and 8283–8606 in the H77 genome, respectively. This is in accord with the criteria in the consensus proposals for the HCV nomenclature [19]. As such, partial sequences in the two regions have accrued, accounting for a major proportion of those archived in various HCV databases, and are now being routinely processed in many laboratories around the world. However, there remains an absence of the molecular rates that exactly match the position and length of these two regions in the HCV genome. In this study, this was also addressed.

## Materials and Methods

### Assembly of sequence datasets

From the Los Alamos HCV database (http://hcv.lanl.gov), 627 full-length genomic sequences of HCV were retrieved. They represented subtypes 1a and 1b and had known sampling dates: i.e. from 1977–2008 for 1a and from 1983–2008 for 1b. To reduce the computational burden while maintaining sufficient temporal (a balance of the sampling dates) and phylogenetic (an even dispersion in the phylogenetic trees) structures, no more than 20 sequences were selected in each year and at least one was retained from each phylogenetic cluster. Thus, two datasets were assembled, one containing 176 sequences of 1a, and the other 144 of 1b (Figure S1 panels A and B and Figure S2). According to the nucleotide numbering in the H77 genome, these sequences were partitioned into nine genomic regions: the Core, E1, E2, P7, NS2, NS3, NS4 (NS4A+NS4B), NS5A and NS5B regions, containing 573, 576, 1,089, 189, 651, 1,893, 945, 1,344 and 1,776 nt, respectively. Since the HVR1 (81nt) and HVR2 (24nt) in E2 [20]–[22], and V3 (72nt) [23] in NS5A are highly variable, while their evolutionary patterns are too complicated to be simulated using the currently available molecular clock models, they were removed. Ultimately, there were 984 nucleotides that remained in E2, 1,272 in NS5A and 8,859 in the entire ORF.

In addition to the nine genomic regions, two routinely amplified regions, the partial Core-E1 and partial NS5B, were also analyzed. For 1a, such sequences were directly trimmed from the ORF dataset because it displayed a sufficient temporal structure (Figure S1 panel A). For 1b, 68 more partial Core-E1 and 160 more partial NS5B sequences were added (Figure S1 panels E and F and Figure S3).

### Clock-likeness analysis of the molecular phylogenies

Before using the nearest neighbor interchange (NNI) perturbation algorithm to heuristically search the maximum likelihood (ML) trees, the best-fitting substitution model was selected using the MEGA5 model test function according to the corrected Akaike Information Criterion (AICc). A regression of root-to-tip genetic distances against the sampling dates was performed using Path-o-gen software (http://tree.bio.ed.ac.uk/software/pathogen). This was done to investigate the clock-likeness of the ML tree based on the simple concept that “distance equals rate multiplied by time” [1]. This is to say that the substitution rate remains constant through time, such the distance equals the genetic distance from the sampled sequences to the root, and time equals the sampling time. The root of the ML tree is chosen to maximize the coefficient of the determinant, R^2^, which measures the clock-likeness of the sequences. The slope of the regression line equals the substitution rate, while the X-intercept is the time for the root node to form, i.e. tMRCA. However, both the substitution rate and the tMRCA obtained from the root-to-tip regression are quite preliminary and inaccurate due to certain limitations, such as: (1) both of these measures are calculated under the assumption of a constant substitution rate, which may not be realistic; (2) it is assumed that the sequences are independent, which may not be true, because they share an evolutionary history and one sequence might even be a direct ancestor of the other; (3) the results are based on a single ML tree, but there is always considerable uncertainty in the estimation of a true tree [1].

### Bayesian MCMC evolutionary analyses

More accurate evolutionary rates and tMRCAs may be estimated using the Bayesian Markov Chain Monte Carlo (MCMC) [24] algorithm implemented in the BEAST software (version 1.6.1). Prior to the MCMC analysis, selections had to be made for each of the substitution, demographic and clock models [11]. For the substitution model, the general time-reversible with invariable-sites-plus-gamma substitution (GTR+I+Γ) model was found to be the best for these datasets and was thus selected for all of the subsequent analyses. For the demographic information, the Bayesian skyline coalescent model was found to always outperform the other four models (i.e. constant size, exponential growth, logistic growth and expansion growth) in previous reports and this was expected to also be the case in the current analysis of HCV sequences [9], [11]. Hence, this model with piecewise-linear population growth was chosen. For the clock model, each of the strict, uncorrelated exponential and uncorrelated lognormal models was tested in combination with the Bayesian skyline model to find the best-fitting one. Because a strict criterion was followed in selecting the HCV sequences, both the 1a and 1b datasets exhibited adequate temporal and phylogenetic structure. This enabled us to estimate the evolutionary rates directly from the two datasets, instead of having to use external ones for calibration, which reduced the bias. As for the prior, we applied a uniform distribution with the lower bound at 0 and the upper bound at 0.01 [25]. The length of the MCMC sampling was initially set for 100 million states and output a tree and a log file every 10,000 states. Using the Tracer software version 1.5 (http://tree.bio.ed.ac.uk), the sampling convergence was assessed by the estimated effective sampling size (ESS). In this study, when all of the ESS numbers were >200, sufficient sampling was considered to have been achieved. However, for those that did not exhibit an adequate ESS number, we extended the chain length of the MCMC procedure up to 300 million states. When the ESS number was still not adequate, we repeated the latter analysis in 300 million states for an additional duplicate and then used the LogCombiner program in the BEAST package to combine the resulting log files for assessing the convergence using Tracer. The Bayesian factor (BF) was computed to compare the paired clock models in order to choose the statistically more rigorous one. Finally, t-test and Mann–Whitney U test in the R program were used to calculate the differences in molecular rates between the analyses.

## Results

### Clock-likeness analysis by regression

**Figure 1:**
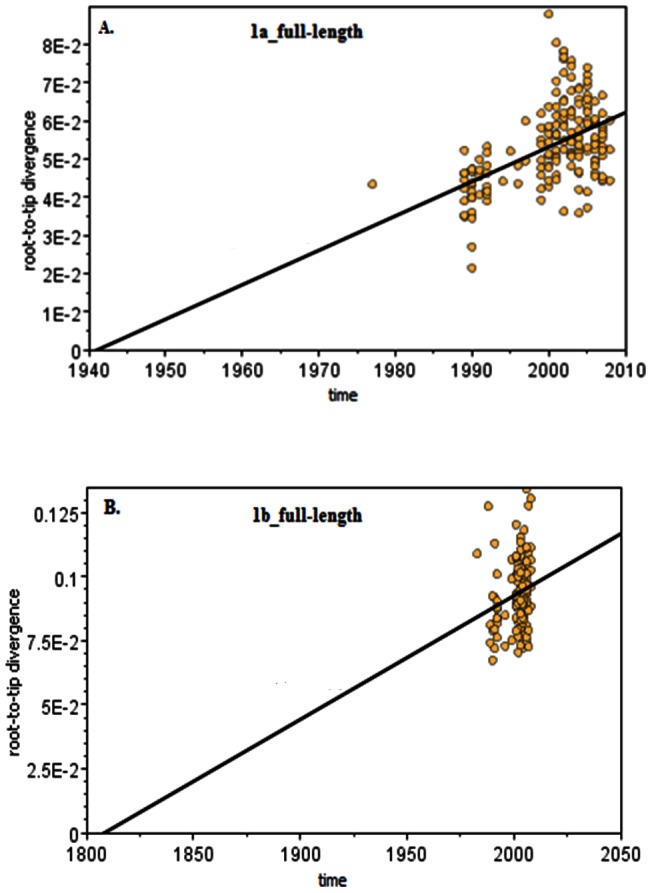
Root-to-tip regression to estimate the tMRCAs and clock rates. A simple linear regression of the root-to-tip genentic distances against the sampling dates was performed using the Path-o-gen software. The root was determined by maximizing the coefficent of determinant R^2^. The vertical axis measures the genetic distances between the samples and the root while the horizontal axis scales the sampling dates (year). For subtype 1a (A), the mean evolutionary rate (the slope of regression line) is 9.05E-4 substitution/site/year and the tMRCA (the X-intercept) is located at 1941. For subtype 1b (B), the mean evolutionary rate is 4.82E-4 and the tMRCA is located at 1808.

Model-testing showed GTR+Γ+I to be the best among the 24 models based on the AICc. Using this model, ML trees were reconstructed and root-to-tip regression analyses were performed (Figure 1). For 1a, the estimated function of linear regression is *d* = 0.0009× (*t*-1941), where *d* is the distance from the samples to the selected root, while *t* is the sampling date. For 1b, the function is *d* = 0.00048× (*t*-1808). The molecular rates and tMRCAs were based on the slope and the X-intercept of the regression lines, respectively. The rates indicated that 1a evolved almost one fold faster and diverged approximately 133 years later than 1b. Root-to-tip regression was also performed for the nine genomic regions (Table 1). Unexpectedly, the molecular rate and the tMRCA estimated for the NS5B dataset of subtype 1b are unrealistic, for the former is negative while the latter occurs in the future, a result that may possibly be ascribed to the stochastic nature of the substitution process. The sequences sampled earlier, exhibiting a greater divergence from the root than those sampled later, may suggest that the evolution of the NS5B region for subtype 1b is not clock-like, or alternatively, that it only reflects one of the limitations of the root-to-tip regression analysis [26]. Regardless, all of the results showed that the nine genomic regions of 1a have a faster evolutionary rate than 1b, consistent with the analyses over the entire ORF and using the BEAST program that were described below.

**Table 1:** The evolutionary rates and tMRCAs estimated for the 1a and 1b datasets in nine genomic regions and over ORF by root-to-tip regression.

1a			1b		
Region	rate	tMRCA	Region	rate	tMRCA¶
core	2.53E-04	1900.439	Core	1.63E-04	1843.535
E1	8.10E-04	1873.517	E1	2.41E-04	1772.206
E2	2.55E-03	1935.909	E2	1.59E-3	1938.742
P7	8.97E-04	1937.205	P7	3.34E-04	1770.422
NS2	1.04E-03	1944.186	NS2	7.92E-04	1897.943
NS3	7.77E-04	1960.988	NS3	2.85E-04	1850.384
NS4	4.70E-04	1919.278	NS4	3.15E-04	1866.972
NS5A	1.24E-03	1935.909	NS5A	9.33E-04	1875.398
NS5B	3.68E-04	1950.525	NS5B*	−0.0002	2210.42
Full-ORF	9.05E-04	1941.156	Full-ORF	4.82E-04	1808.093

¶ In the calendar year. * The evolutionary rate is negative and tMRCA locates in the future.

### Bayesian MCMC evolutionary analysis

Most of the analyses exhibited an adequate ESS number for all the statistics resulted, after running the MCMC procedure for 100–300 million states. However, for the 1a and 1b ORF datasets under the exponential model, three analyses each 300 million states were required. After combining their resulting log files, sufficient ESS numbers were finally obtained (Table S1 and S2).

#### Subtype 1a

**Figure 2:**
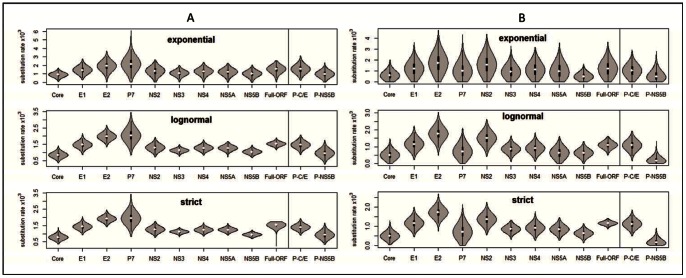
The violin plots of the posterior evolutionary rate estimated using the uniform (0, 0.01) rate prior in the nine genomic regions and over the entire ORF of the subtype 1a (A panel) and 1b (B panel) datasets. Combined with the GTR+I+Γ substitution model and Bayesian skyline coalesent model, the MCMC procedures were run under three clock models, exoponetial, lognormal, and strict, respectively, using BEAST. The vertical axis measures the substitution rate multiplied by 10^−3^ (substitution/site/year). The horizontal axis indicates the nine genomic regions and the entire ORF. The left three panels show the results for the 1a dataset. The right three panels show the results for the 1b dataset. In each panel, two violins are separated in a small case on the right, which indicate the rates estimated for the routinely amplified partial Core-E1 (P-C/E1) and partial NS5B (P-NS5B) regions.

**Figure 3:**
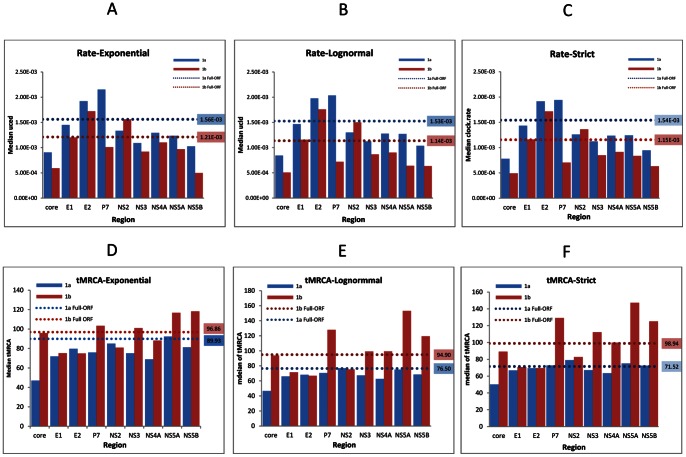
The median evolutionary rates and the tMRCAs estimated in the nine genomic regions and over the entire ORF of the subtype 1a and 1b datasets. Panels A, B, and C show the median evolutionary rates. Panels D, E, and F show the median tMRCAs. The blue columns represent the estimates for 1a. The red columns represent the estimates for 1b. The dash lines indicate the estimates for the entire ORF.

To demonstrate the influence of the uniform rate prior on the posterior rates estimated under the three models for the different datasets, we plotted the marginal posterior rate densities as violin plots (Figure 2). A violin plot is a combination of a box plot and a rotated kernel density curve to display the probability density of a given parameter [25], [27]. In this study, except for the ORF under the strict model, all of the 1a marginal posterior rate density curves are bell shaped. Combined with the information in Table S1, the three models estimated very close median rates for a given dataset. However, the differences in their 95% confidence intervals are quite large, the largest being that under the exponential model and the smallest under the strict. The Core region exhibited the lowest median rates (9.04×10^−4^, 8.43×10^−4^, and 7.78×10^−4^ under the exponential, lognormal, and strict models, respectively), while the P7 the largest (2.15×10^−3^, 2.04×10^−3^ and 1.94×10^−3^), which means that the P7 region has evolved more than two times faster than the Core. The rank of the median rates, P7>E2>E1>NS2>NS4>NS5A>NS3>NS5B>Core, was consistently obtained with the three models. In addition, the ORF exhibited consistent median rates (1.56×10^−3^ in exponential, 1.53×10^−3^ in lognormal and 1.55×10^−3^ in strict) under the three models, which are the most close to those given under the same model for the E1 region (1.45×10^−3^ in exponential, 1.47×10^−3^ in lognormal and 1.43×10^−3^ in strict). However, the rate heterogeneity among sites (α) is variable among datasets. A high α value suggests a weak mutational “hot spot” [28]. Compared with the values provided by the root-to-tip regression, higher and more accurate rates were estimated using the MCMC procedure. In contrast to the median rates, the median tMRCAs were found to be largely similar across different genomic regions, particularly under the strict model (Figure 3). Theoretically, the rates are variable, but the ages estimated by the tMRCAs are identical. This is because even though they estimate the ancestor based on different genomic regions, they refer to the same ancestor. Therefore, the degree to which tMRCAs differ among the regions can be used to evaluate the robustness of the MCMC process. Bayes Factor comparison showed that the exponential model outperformed the other two models in all of the nine genomic regions. For the ORF dataset, however, the lognormal is the best model (Table S1).

#### Subtype 1b

Among the nine genomic regions, E2 displayed the highest median rates in all of the three models (1.72×10^−3^ in the exponential, 1.76×10^−3^ in the lognormal, and 1.71×10^−3^ in the strict). In contrast, the Core exhibited the lowest median rates in the lognormal (5.06×10^−4^) and strict (4.91×10^−4^), while the NS5B had the lowest median rate (4.47×10^−4^) in the exponential. The P7 median rates were lower than those in some other regions, although they were the highest for 1a under all of the three models. In the exponential model, the rank of the median rates in the nine genomic regions turned out to be E2>NS2>E1>NS4>P7>NS5A>NS3>Core>NS5B, while under the other two models, the rank was slightly different, i.e. E2>NS2>E1>NS4>NS3>P7>NS5A>NS5B>Core in the lognormal and E2>NS2>E1>NS4>NS3>NS5A>P7>NS5B>Core in the strict. To a greater extent than for 1a, the 1b ORF exhibited the median rates (1.21×10^−3^ in exponential, 1.14×10^−3^ in lognormal and 1.15×10^−3^ in strict) that are highly similar to that in the E1 region under the same models, which can be a piece of strong evidence to indicate that the E1 region can best represent the entire ORF for estimating the molecular rates. Not all of the marginal posterior rate densities are bell shaped, especially for those obtained with the exponential model (Figure 2). Vastly different median tMRCAs were estimated for the nine genomic regions (Figure 3). For example, in the lognormal model, the median tMRCA estimated for the NS5A region indicated that the divergence of 1b took place approximately 153 years ago, while the tMRCA given for the E2 region showed the divergence of 1b occurring approximately 67 years ago. Bayes Factor comparison showed that the exponential model is the best for the Core, E1, P7, NS2, NS4 and NS5A regions, while the lognormal model is the best for E2, NS3, NS5B and the entire ORF dataset (Table S2).

### Comparison of 1a and 1b

Excluding NS2, 1a exhibited higher median rates than 1b in eight genomic regions under all of the three models (Table S1 and S2). T-test and Mann–Whitney U test revealed significant differences (p values <0.0001) for their median rates and median tMRCAs.

Because the tMRCAs for 1b are less robust than for 1a, 17 E1 sequences of 1a sampled in 1981–1998 and 20 E1 sequence of 1b sampled in 1982–2000 were added to the datasets for a reanalysis under the exponential model (Figure S1 panel C and D and Figure S4). To determine the level of bias, T-test was performed to compare the rate resulting from the reanalysis with the rate resulting from the original dataset. For 1a, we tested the null hypothesis that the difference between these two rates is larger than 5% of the original rate, i.e. the difference is greater than 7.24×10^−5^ (reanalysis rate  = 1.478×10^−3^, original rate  = 1.448×10^−3^). After testing, a p-value of <0.001 was obtained, which rejects the hypothesis. Moreover, for the 1b dataset the null hypothesis is that the difference between the rate from the reanalysis and the original rate is less than 15% of the original rate, i.e. the difference is less than 1.8×10^−4^ (reanalysis rate  = 1.52×10^−3^, original rate  = 1.20×10^−3^). With a 0.00032 p-value, the hypothesis is rejected again. Jointly, these results confirm that a significantly greater rate change was shown by 1b than 1a when more sequences were included for a reanalysis and that the rates estimated for 1a are more robust than for 1b.

### Analysis of sequences in two routinely amplified regions

#### Subtype 1a

For the partial Core-E1 region, the median rates were 1.56×10^−3^, 1.47×10^−3^ and 1.40×10^−3^ in the exponential, lognormal, and strict models, respectively, while for the partial NS5B the median rates were 9.77×10^−4^, 9.54×10^−4^ and 9.26×10^−4^. These rates all exhibited bell-shaped distributions (Figure 2A) with a satisfactory ESS number after the MCMC analyses each was run for 100 million chain length. Bayesian Factor comparison showed that the exponential model is the best for both regions. Similar to that above described, the three Core-E1 rates are close to those estimated for the ORF under the same models (Table S1).

#### Subtype 1b

For the partial Core-E1 region, the median rates are 1.02×10^−3^, 1.08×10^−2^ and 1.12×10^−3^ in the exponential, lognormal, and strict models, respectively, while for the partial NS5B the rates are 4.85×10^−4^, 1.69×10^−4^ and 1.50×10^−4^. They all exhibit satisfied ESS numbers. The Core-E1 rates are highly similar to each other and their distribution patterns are bell-shaped (Figure 2B). However, the NS5B rates are very low and their distribution patterns are truncated. This implies that the NS5B rates are not robust. Bayesian Factor comparison showed that the exponential model is the best for the partial Core-E1 while the lognormal is the best for the partial NS5B (Table S2).

## Discussion

This report is one of a few evolutionary studies that have been performed based on the BEAST analysis of the full-length HCV genomic sequences. According to the organization of the HCV genome, the sequences were partitioned into nine genomic regions that were analyzed individually or over the entire ORF. The evolutionary information, such as the molecular rates and the tMRCAs were systematically provided. It should be noted that all the posterior information was obtained based on a uniform distribution of the evolutionary rate prior in a range from 0 to 0.01. This approach was used due to the lack of any additional information, except for the dated sequences that are the major factor used to generate all the posteriors.

Recently, Gray et al. [18] used a mathematical partition approach to analyze the full-length genomic sequences of HCV subtypes 1a and 1b. They split the whole genome into 21 non-overlapping equal-length partitions and estimated the evolutionary rate for each partition. This was the first quantification of the variation in HCV evolutionary dynamics at different scales. However, our analyses are somehow different because the following factors were taken into account. As is well known, the HVR1 and HVR2 of the HCV genome are exposed to strong positive selection by the host immune responses, which individualize the two regions and make them inconsistent with the neutral theory and molecular clock rule [29], [30]. In this case, the currently proposed molecular clock models (the strict, exponential, lognormal and random local clock models) may not be able to precisely simulate their evolutionary modes. A similar case may also exist for the V3 region of NS5A, because its related gene products have been found to closely interact with the HCV envelope proteins [31]. On the other hand, HCV evolution may have suffered from constraints on base changes due to negative selection. For example, the HCV genome is known to be highly ordered, forming complex RNA secondary structures throughout the genome, which has been termed “genome-scale ordered RNA structure” (GORS) [32]. The requirement for base-pairing in such a GORS structure severely limits the potential for independent evolution of a single mathematical partition from its flanking ones. Furthermore, the HCV genome is comprised of a single ORF consisting of four structural (Core, E1, E2 and P7) and six nonstructural regions (NS2, NS3, NS4A, NS4B, NS5A and NS5B). From this ORF, a polyprotein is first translated, followed by processing into a number of proteins corresponding to the different genomic regions; meanwhile, to complete the life cycle of the virus, each of these proteins serves as a unique and indispensable component [33]. Following the molecular rule, we removed the 5′- and 3′-UT regions, HVR1 and HVR2 in the E2 region, and V3 in the NS5A region. Based on a biological dissection of the HCV genome, we partitioned the 1a and 1b ORF datasets into nine genomic regions. We therefore provided the information on the HCV evolutionary rates from a different aspect.

To obtain sufficient temporal and spatial structures, the 1a and 1b datasets were carefully checked for a good balance of sampling dates and an even dispersion in phylogenetic trees. Based on the best-fitting substitution model and three different clock models, a set of posterior evolutionary rates were estimated for each of the nine genomic regions and over the ORF. Higher rates were observed in the E2 and P7 regions, while lower rates were seen in the Core and NS5B. In addition, most of the genomic regions of subtype 1a exhibited significantly higher rates than their counterparts of 1b. These results demonstrate that the HCV evolutionary rates are different among the various subtypes and genomic regions. With these results, a rule is indicated that a realistic estimation of HCV epidemic history requires a right selection of the rate prior, which should match the actual dataset for fitting the subtype and genomic region, as well as the right sequence length.

In this study, the existence of more robust 1a evolutionary rates than 1b was indicated by a series of highly credible tMRCAs. Theoretically, the tMRCAs estimated for different partitions of the same dataset should be identical, because they refer to the same ancestors. Here, the median tMRCAs estimated for the nine genomic regions of the 1a dataset are very close, but they are slightly smaller than that for the ORF (Figure 3). These results suggest that the MCMC procedures and the generated rates for the nine genomic regions are reliable. In contrast, the median tMRCAs estimated for the 1b dataset are diverse, which diversity is likely attributable to its smaller sample size and weaker temporal structure. In the 1b dataset, 17.36% of the sequences were sampled in 1983–1999 and 82.64% in 2000–2007, while in the 1a dataset these percentages were 28.41% and 71.02%, respectively. The weaker temporal structure in the 1b dataset was also indicated after including additional E1 sequences for reanalysis. In the reanalysis, 17 E1 sequences of 1a sampled in 1981–1998 and 20 E1 sequences of 1b in 1982–2000 were added to the corresponding dataset. The BEAST analyses generated a new 1a rate that is no more than 5% larger than the original 1a rate. However, the difference in the 1b rates was greater than 15%. These results demonstrate that a good temporal structure is critical for a precise estimation of the evolutionary rate. It also implies that the statistics generated with this 1b dataset might have certain biases and thus should be used with caution until a better one is assembled that contains more 1b sequences sampled before 2000. In addition, the weaker rates estimated for the 1b dataset were also suggested by the rate distribution shapes which appeared in the violin plots. The latter graphically illustrate the mean, variance and density curve of estimates and provide additional information for the BEAST analysis. These violin plots revealed that all of the 1a rate marginal densities were bell shaped, while some of the densities for 1b, especially those based on the exponential model, displayed truncated or poorly formed bell shapes (Figure 2). That 1a has a higher evolutionary rates than 1b is consistent with the results from a recent report, in which this pattern ascribed to the difference in the 1a and 1b transmission modes [18]. While in that report only 63 subtype 1a and 54 subtype 1b sequences were used in order to reduce the computation burden, the estimated evolutionary rates were 1.44–1.48×10^−3^ substitutions/site/year for 1a and 1.18–1.25×10^−3^ for 1b over the full genome length, which are only slightly different from the median rates 1.53–1.56×10^−3^ for 1a and 1.14–1.25×10^−3^ for 1b that we estimated under similar conditions.

One more point worth noting is that the current HCV classification and nomenclature are largely based on the characterization of partial sequences in the Core-E1 and NS5B regions [19]. Therefore, in various HCV databases, these sequences comprised the majority. Although in previous studies two evolutionary rates, 7.9×10^−4^ substitutions/site/year for E1 and 5.0×10^−4^ for the NS5B region, have been effectively used as priors, there remains the need to estimate the rates in a manner that exactly matches the size of these two routinely amplified regions [12]. Based on the more accurate sizes trimmed from the dataset of 176 1a sequences, we estimated relatively consistent rates under three clock models, 1.40–1.56×10^−3^ substitutions/site/year for the partial Core-E1 and 9.26–9.77×10^−4^ for the partial NS5B region. Using a similar strategy but including more 1b sequences, we estimated lower median rates between 1.02–1.12×10^−3^ for the partial Core-E1 and much lower median rates between 1.50–4.85×10^−4^ for the partial NS5B. A noteworthy finding is that excluding the rates for the partial NS5B of 1b, all the other median rates are almost twice as high as those previously reported. Therefore, the question arises as to whether a lower rate to estimate the dynamics of HCV would result in an underestimation of the speed of HCV evolution and thus indicate an earlier timing for the estimated HCV growth and the correlated historical events. In addition, it was revealed that the partial Core-E1 region can substantially represent the ORF for estimating the HCV rates, although to a less extent than the E1 region that did under the same models. From a different point of view, this finding helps explain why the partial Core-E1 region is preferably used for the HCV classification.

## Supporting Information

Figure S1 **Histograms to exhibit the temporal structure of the six sequence datasets: (A) the 176 full-length sequences of subtype 1a, (B) the 144 full-length sequences of subtype 1b, (C) the 193 E1 region sequences of subtype 1a, assembled by adding 17 E1 region sequences to the 176 taxa of (A), (D) the 164 E1 region sequences of subtype 1b, assembled by adding 20 E1 region sequences to the 144 taxa of (B), (E) the 212 partial Core-E1 region sequences of subtype 1b, assembled by adding 68 partial Core-E1 sequences to the 144 taxa of (B), and (F) the 304 partial NS5B region sequences of subtype 1b, assembled by adding 160 partial NS5B sequences to the 144 taxa of (B).** In each diagram, the vertical axis measures the number of sequences and the horizontal axis scales the year when the sequences were sampled.
(PNG)

Figure S2 **Two ML trees to show the phylogenetic dispersion of: (A) the 176 full-length sequences of subtype 1a, and (B) the 144 full-length sequences of subtype 1b.** Each tip of tree represents one sequence that is indicated with its sampling year followed by its Genbank accession number. A ruler under each tree measures the substitution per nucleotide site.
(PPTX)

Figure S3 **Two ML trees to show the phylogenetic dispersion of: (A) the 212 partial Core-E1 region sequences, and (B) the 304 partial NS5B region sequences.** They all belong to subtype 1b. The black branches represent those trimmed from the (B) dataset shown in Figure S2. The red branches indicate those retrieved from the Los Alamos HCV database. We added these sequences in order to increase the balance of the temporal structure and even dispersion in phylogenetic tree. Otherwise, all of the indications remain the same as that described above for Figure S2.
(PPTX)

Figure S4 **Two ML trees to show the phylogenetic dispersion of: (A) the 193 E1 region sequences of subtype 1a, and (B) the 166 E1 region sequences of subtype 1b.** The black branches in the respective dataset represent those trimmed from the dataset (A) or (B) shown in Figure S2. The red branches indicate those added for subtype 1a (17 isolates) or 1b (20 isolates), for which only partial sequences are available in the Los Alamos HCV database. We added these sequences in order to increase the balance of the temporal structure and even dispersion in phylogenetic tree. Otherwise, all of the indications remain the same as that described above for Figure S2.
(PPTX)

Table S1 **The related statistics (mean ± Stderr) generated in the Bayesian MCMC analysis of the subtype 1a dataset.**
(DOCX)

Table S2 **The related statistics (mean ± Stderr) generated in the Bayesian MCMC analysis of the subtype 1b dataset.**
(DOCX)
