# Supplementary figures and images for: The Evolutionary Rates of HCV Estimated with Subtype 1a and 1b Sequences over the ORF Length and in Different Genomic Regions
Source: PLoS One. 2013 Jun 6;8(6):e64698. doi: 10.1371/journal.pone.0064698 (PMC3675120; doi:10.1371/journal.pone.0064698)

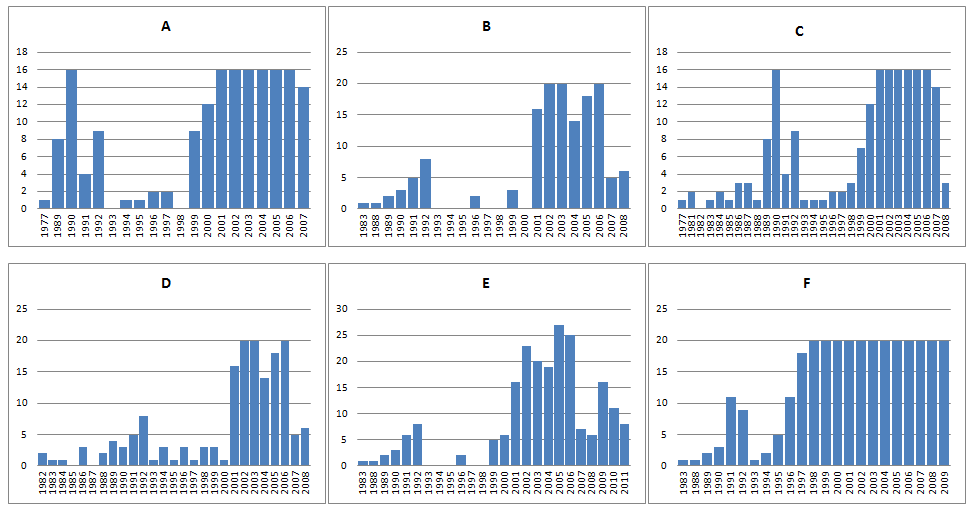

Supplement: Figure S1 — Histograms to exhibit the temporal structure of the six sequence datasets: (A) the 176 full-length sequences of subtype 1a, (B) the 144 full-length sequences of subtype 1b, (C) the 193 E1 region sequences of subtype 1a, assembled by adding 17 E1 region sequences to the 176 taxa of (A), (D) the 164 E1 region sequences of subtype 1b, assembled by adding 20 E1 region sequences to the 144 taxa of (B), (E) the 212 partial Core-E1 region sequences of subtype 1b, assembled by adding 68 partial Core-E1 sequences to the 144 taxa of (B), and (F) the 304 partial NS5B region sequences of subtype 1b, assembled by adding 160 partial NS5B sequences to the 144 taxa of (B). In each diagram, the vertical axis measures the number of sequences and the horizontal axis scales the year when the sequences were sampled. (PNG) [file pone.0064698.s001.png]

## Slide 1
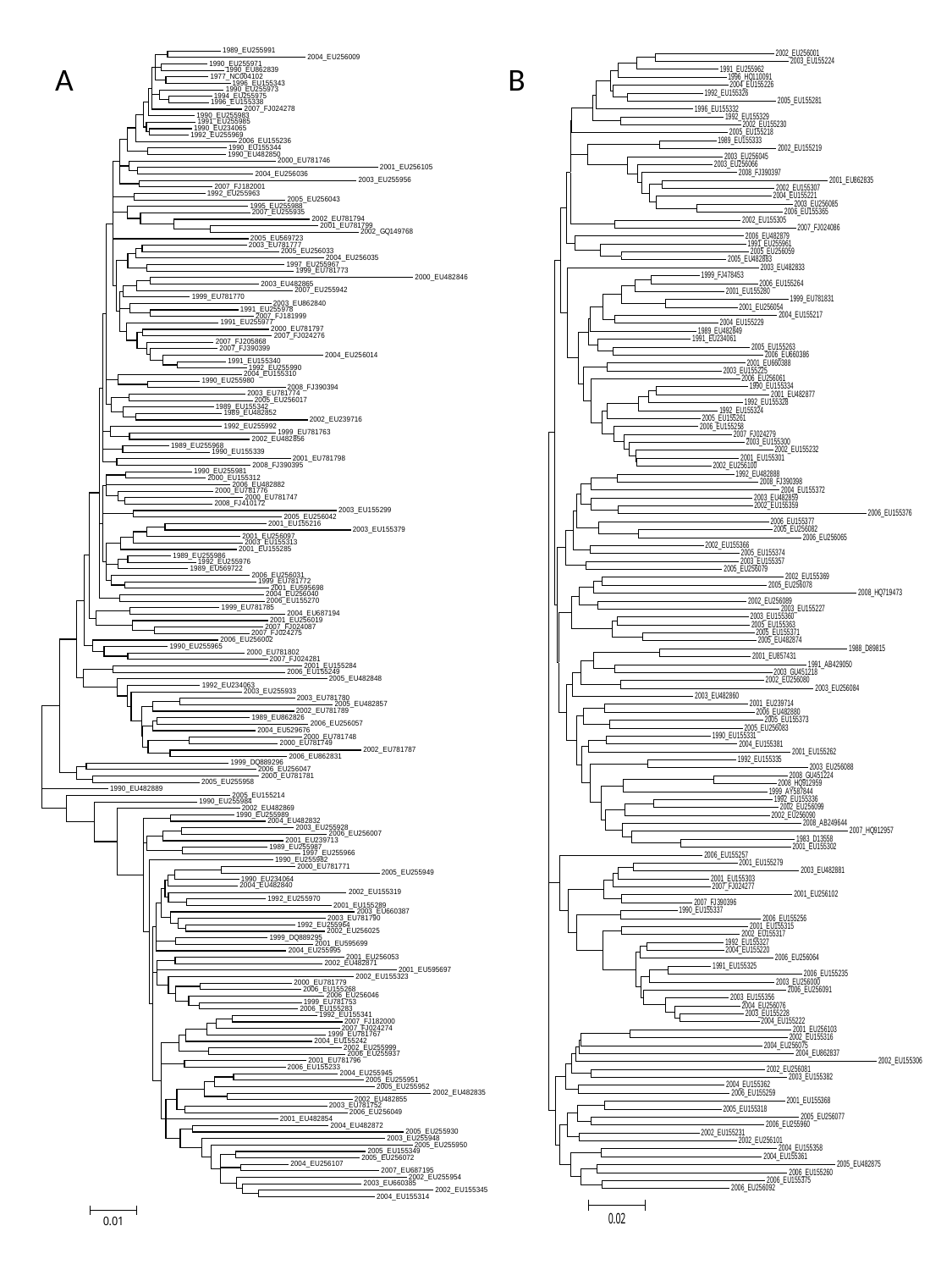

A
B

Supplement: Figure S2 — Two ML trees to show the phylogenetic dispersion of: (A) the 176 full-length sequences of subtype 1a, and (B) the 144 full-length sequences of subtype 1b. Each tip of tree represents one sequence that is indicated with its sampling year followed by its Genbank accession number. A ruler under each tree measures the substitution per nucleotide site. (PPTX) [file pone.0064698.s002.pptx]
