# Supplementary material for: The Evolutionary Rates of HCV Estimated with Subtype 1a and 1b Sequences over the ORF Length and in Different Genomic Regions
Source: PLoS One. 2013 Jun 6;8(6):e64698. doi: 10.1371/journal.pone.0064698 (PMC3675120; doi:10.1371/journal.pone.0064698)
Supplement: Figure S3 — Two ML trees to show the phylogenetic dispersion of: (A) the 212 partial Core-E1 region sequences, and (B) the 304 partial NS5B region sequences. They all belong to subtype 1b. The black branches represent those trimmed from the (B) dataset shown in Figure S2. The red branches indicate those retrieved from the Los Alamos HCV database. We added these sequences in order to increase the balance of the temporal structure and even dispersion in phylogenetic tree. Otherwise, all of the indications remain the same as that described above for Figure S2. (PPTX) [file pone.0064698.s003.pptx]

## Slide 1
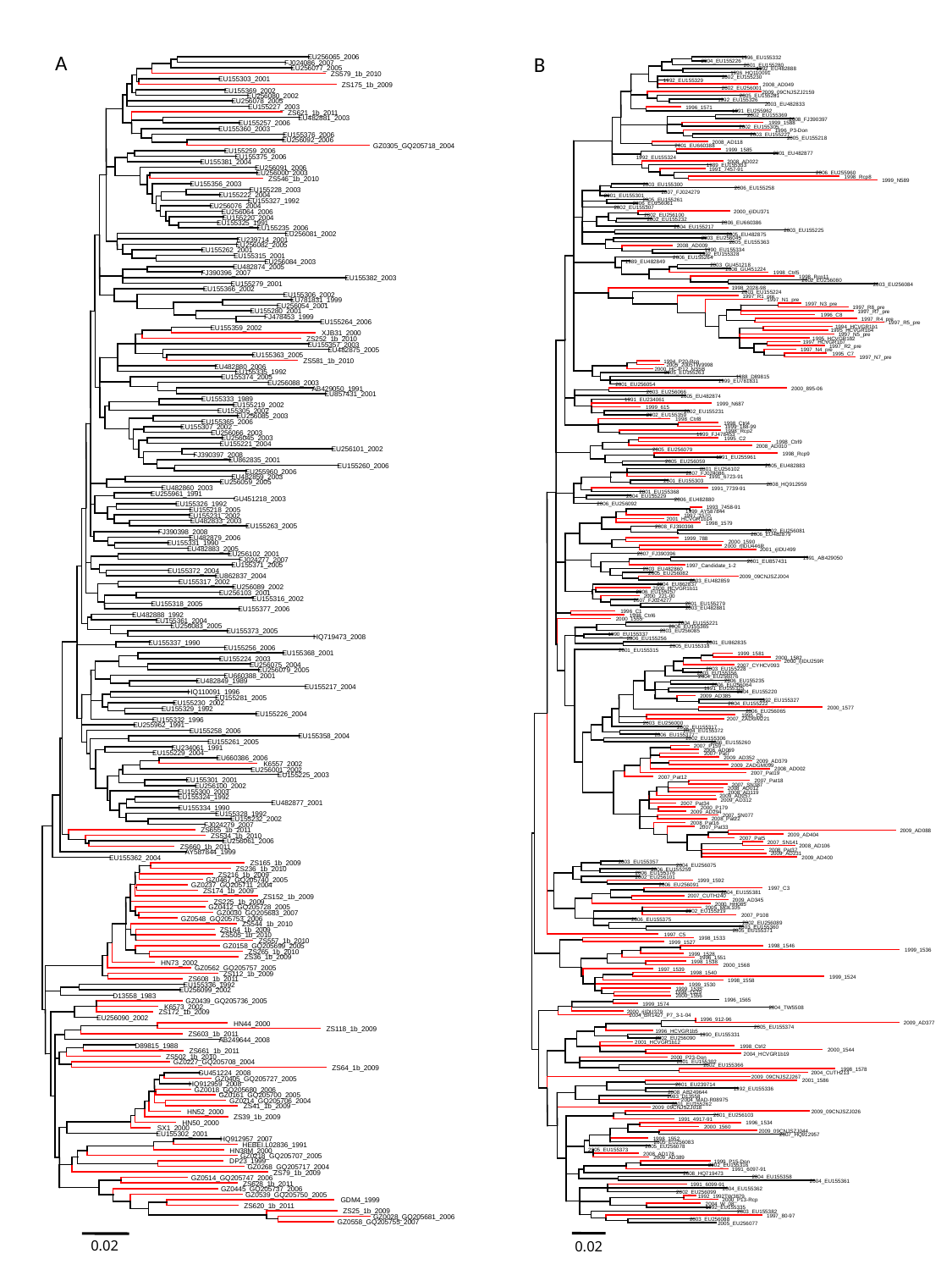

A
B
 EU256065_2006
 FJ024086_2007
 EU256077_2005
 ZS579_1b_2010
 EU155303_2001
 ZS175_1b_2009
 EU155369_2002
 EU256080_2002
 EU256078_2005
 EU155227_2003
 ZS621_1b_2011
 EU482881_2003
 EU155257_2006
 EU155360_2003
 EU155376_2006
 EU256092_2006
 GZ0305_GQ205718_2004
 EU155259_2006
 EU155375_2006
 EU155381_2004
 EU256091_2006
 EU256000_2003
 ZS546_1b_2010
 EU155356_2003
 EU155228_2003
 EU155222_2004
 EU155327_1992
 EU256076_2004
 EU256064_2006
 EU155220_2004
 EU155325_1991
 EU155235_2006
 EU256081_2002
 EU239714_2001
 EU256082_2005
 EU155262_2001
 EU155315_2001
 EU256084_2003
 EU482874_2005
 FJ390396_2007
 EU155382_2003
 EU155279_2001
 EU155366_2002
 EU155306_2002
 EU781831_1999
 EU256054_2001
 EU155280_2001
 EU256045_2003
 EU155221_2004
 EU256101_2002
 FJ390397_2008
 EU862835_2001
 EU155260_2006
 EU255960_2006
 EU482859_2003
 EU256059_2005
 EU482860_2003
 EU255961_1991
 GU451218_2003
 EU155326_1992
 EU155218_2005
 EU155231_2002
 EU482833_2003
 EU155263_2005
 FJ390398_2008
 EU482879_2006
 EU155331_1990
 EU482883_2005
 EU256102_2001
 FJ024277_2007
 EU155371_2005
 EU155372_2004
 EU862837_2004
 EU155317_2002
 EU256089_2002
 EU256103_2001
 EU155316_2002
 EU155318_2005
 EU155377_2006
 EU482888_1992
 EU155361_2004
 EU256083_2005
 EU155373_2005
 HQ719473_2008
 EU155337_1990
 EU155256_2006
 EU155368_2001
 EU155224_2003
 EU256075_2004
 EU256079_2005
 EU660388_2001
 EU482849_1989
 EU155217_2004
 HQ110091_1996
 EU155281_2005
 EU155230_2002
 EU155329_1992
 EU155226_2004
 EU155332_1996
 EU255962_1991
 EU155258_2006
 EU155358_2004
 EU155261_2005
 EU234061_1991
 EU155229_2004
 EU660386_2006
 K6557_2002
 EU256001_2002
 EU155225_2003
 EU155301_2001
 EU256100_2002
 EU155300_2003
 EU155324_1992
 EU482877_2001
 EU155334_1990
 EU155328_1992
 EU155232_2002
 FJ024279_2007
 ZS655_1b_2011
 ZS534_1b_2010
 EU256061_2006
 ZS660_1b_2011
 AY587844_1999
 EU155362_2004
 ZS165_1b_2009
 ZS236_1b_2010
 ZS216_1b_2009
 GZ0467_GQ205740_2005
 GZ0237_GQ205711_2004
 ZS174_1b_2009
 ZS152_1b_2009
 ZS225_1b_2009
 GZ0412_GQ205728_2005
 GZ0030_GQ205683_2007
 GZ0548_GQ205753_2006
 ZS544_1b_2010
 ZS164_1b_2009
 ZS505_1b_2010
 ZS557_1b_2010
 GZ0158_GQ205699_2005
 ZS265_1b_2010
 ZS36_1b_2009
 HN73_2002
 GZ0562_GQ205757_2005
 ZS112_1b_2009
 ZS608_1b_2011
 EU155336_1992
 EU256099_2002
 D13558_1983
 GZ0439_GQ205736_2005
 K6573_2002
 ZS172_1b_2009
 EU256090_2002
 HN44_2000
 ZS118_1b_2009
 ZS603_1b_2011
 AB249644_2008
 D89815_1988
 ZS661_1b_2011
 ZS502_1b_2010
 GZ0227_GQ205708_2004
 ZS64_1b_2009
 GU451224_2008
 GZ0405_GQ205727_2005
 HQ912959_2008
 GZ0018_GQ205680_2006
 GZ0161_GQ205700_2005
 GZ0214_GQ205706_2004
 ZS41_1b_2009
 HN52_2000
 ZS39_1b_2009
 HN50_2000
 SX1_2000
 EU155302_2001
 HQ912957_2007
 HEBEI.L02836_1991
 HN38M_2000
 GZ0218_GQ205707_2005
 DP23_1999
 GZ0268_GQ205717_2004
 ZS79_1b_2009
 FJ478453_1999
 EU155264_2006
 EU155359_2002
 XJB31_2000
 ZS252_1b_2010
 EU155357_2003
 EU482875_2005
 EU155363_2005
 ZS581_1b_2010
 EU482880_2006
 EU155335_1992
 EU155374_2005
 EU256088_2003
 AB429050_1991
 EU857431_2001
 EU155333_1989
 EU155219_2002
 EU155305_2002
 EU256085_2003
 EU155365_2006
 EU155307_2002
 EU256066_2003
 GZ0514_GQ205747_2006
 ZS628_1b_2011
 GZ0445_GQ205737_2006
 GZ0539_GQ205750_2005
 GDM4_1999
 ZS620_1b_2011
 ZS25_1b_2009
 GZ0028_GQ205681_2006
 GZ0558_GQ205755_2007
0.02
 1996_EU155332
 2004_EU155226
 2001_EU155280
 1992_EU482888
 1996_HQ110091
 2002_EU155230
 1992_EU155329
 2008_AD049
 2002_EU256001
 2009_09CNJSZJ2159
 2005_EU155281
 1992_EU155326
 2003_EU482833
 1996_1571
 1991_EU255962
 2002_EU155369
 2008_FJ390397
 1999_1588
 2002_EU155305
 1996_P3-Don
 2003_EU155227
 2005_EU155218
 2008_AD118
 2001_EU660388
 1999_1585
 2001_EU482877
 1992_EU155324
 2008_AD022
 1989_EU155333
 1991_7457-91
 2006_EU255960
 1998_Rcp8
 1999_N589
 2003_EU155300
 2006_EU155258
 2007_FJ024279
 2001_EU155301
 2005_EU155261
 2006_EU256061
 2002_EU155307
 2000_rjIDU371
 2002_EU256100
 2002_EU155232
 2006_EU660386
 2004_EU155217
 2003_EU155225
 2005_EU482875
 2003_EU256045
 2005_EU155363
 2008_AD009
 1990_EU155334
 1992_EU155328
 2006_EU155264
 1989_EU482849
 2003_GU451218
 2008_GU451224
 1998_Ctrl5
 1998_Rcp11
 2002_EU256080
 2003_EU256084
 1998_2028-98
 2003_EU155224
 1997_R1_pre
 1997_N1_pre
 1997_N3_pre
 1997_R8_pre
 1997_R7_pre
 1996_C8
 1997_R4_pre
 1997_R5_pre
 1994_HCVGR1b1
 1995_HCVGR1b4
 1997_N5_pre
 1995_HCVGR1b2
 1997_HCVGR1b7
 1997_R2_pre
 1997_N4_pre
 1995_C7
 1997_N7_pre
 1994_P20-Rcp
 2005_2005TW9998
 2000_HC-P12_NS5B
 2005_EU155263
 1988_D89815
 1999_EU781831
 2001_EU256054
 2000_895-06
 2003_EU256066
 2005_EU482874
 1991_EU234061
 1999_N687
 1999_615
 2002_EU155231
 2002_EU155359
 1998_Ctrl8
 1998_Ctrl4
 1999_188-99
 1998_Rcp2
 1999_FJ478453
 1995_C2
 1998_Ctrl9
 2008_AD010
 2005_EU256079
 1998_Rcp9
 1991_EU255961
 2005_EU256059
 2005_EU482883
 2001_EU256102
 2007_FJ024086
 1991_6723-91
 2001_EU155303
 2008_HQ912959
 1991_7739-91
 2001_EU155368
 2004_EU155229
 2006_EU482880
 2006_EU256092
 1993_7458-91
 1999_AY587844
 1997_1570
 2001_HCVGR1b14
 1998_1579
 2008_FJ390398
 2002_EU256081
 2006_EU482879
 1999_788
 2000_1590
 2000_rjIDU446R
 2001_rjIDU499
 2007_FJ390396
 1991_AB429050
 2001_EU857431
 1997_Candidate_1-2
 2003_EU482860
 2005_EU256082
 2009_09CNJSZJ004
 2003_EU482859
 2004_EU862837
 2000_HCVGR1b11
 2006_EU155257
 2000_221-00
 2007_FJ024277
 2001_EU155279
 2003_EU482881
 1996_C1
 1998_Ctrl6
 2000_1555
 2004_EU155221
 2006_EU155365
 2003_EU256085
 1990_EU155337
 2006_EU155256
 2001_EU862835
 2005_EU155318
 2001_EU155315
 1999_1581
 2000_1582
 2000_rjIDU259R
 2007_CYHCV093
 2003_EU155228
 2003_EU155356
 2004_EU256076
 2006_EU155235
 2006_EU256064
 1991_EU155325
 2004_EU155220
 2009_AD385
 1992_EU155327
 2004_EU155222
 2000_1577
 2006_EU256065
 1995_C6
 2007_ZADGM221
 2003_EU256000
 2002_EU155317
 2004_EU155372
 2006_EU155377
 2002_EU155306
 2006_EU155260
 2007_P159
 2008_AD069
 2007_Pat7
 2009_AD352
 2009_AD379
 2009_ZADGM099
 2008_AD002
 2007_Pat19
 2007_Pat12
 2007_Pat18
 2007_SN387
 2008_AD012
 2008_AD119
 2009_AD257
 2009_AD312
 2007_Pat34
 2000_P179
 2009_AD294
 2007_SN077
 2008_Pat22
 2008_Pat16
 2007_Pat33
 2009_AD088
 2009_AD404
 2007_Pat5
 2007_SN141
 2008_AD106
 2008_Pat37
 2009_AD231
 2009_AD400
 2003_EU155357
 2004_EU256075
 2006_EU155259
 2006_EU155376
 2002_EU256101
 1999_1592
 2006_EU256091
 1997_C3
 2004_EU155381
 2007_CUTH240
 2009_AD345
 2000_HH085
 2005_MGL105
 2002_EU155219
 2007_P108
 2006_EU155375
 2002_EU256089
 2003_EU155360
 2005_EU155371
 1997_C5
 1998_1533
 1999_1527
 1998_1546
 1999_1536
 1999_1528
 1996_1551
 1998_1538
 2000_1568
 1997_1539
 1998_1540
 1999_1524
 1998_1558
 1999_1530
 1999_1535
 1998_1529
 2000_1556
 1996_1565
 1999_1574
 2004_TW5508
 2000_rjIDU379
 2004_BR1427_P7_3-1-04
 1996_912-96
 2009_AD377
 2005_EU155374
 1996_HCVGR1b5
 1990_EU155331
 2002_EU256090
 2001_HCVGR1b12
 1998_Ctrl2
 2000_1544
 2004_HCVGR1b19
 2000_P23-Don
 2001_EU155302
 2002_EU155366
 1998_1578
 2004_CUTH213
 2009_09CNJSZJ267
 2001_1586
 2001_EU239714
 1992_EU155336
 2008_AB249644
 1983_D13558
 2004_MAD-R08975
 2001_EU155262
 2009_09CNJSZJ018
 2009_09CNJSZJ026
 2001_EU256103
 1991_4917-91
 1996_1534
 2000_1560
 2009_09CNJSZJ044
 2007_HQ912957
 1998_1552
 2005_EU256083
 2005_EU256078
 2005_EU155373
 2008_AD178
 2009_AD389
 1999_P15-Don
 2002_EU155316
 1991_6097-91
 2008_HQ719473
 2004_EU155358
 2004_EU155361
 1991_6099-91
 2004_EU155362
 2002_EU256099
 1992_1992TW3879
 2000_P13-Rcp
 2004_W_08
 1992_EU155335
 2003_EU155382
 1997_80-97
 2003_EU256088
 2005_EU256077
0.02
