# Supplementary material for: The Evolutionary Rates of HCV Estimated with Subtype 1a and 1b Sequences over the ORF Length and in Different Genomic Regions
Source: PLoS One. 2013 Jun 6;8(6):e64698. doi: 10.1371/journal.pone.0064698 (PMC3675120; doi:10.1371/journal.pone.0064698)
Supplement: Figure S4 — Two ML trees to show the phylogenetic dispersion of: (A) the 193 E1 region sequences of subtype 1a, and (B) the 166 E1 region sequences of subtype 1b. The black branches in the respective dataset represent those trimmed from the dataset (A) or (B) shown in Figure S2. The red branches indicate those added for subtype 1a (17 isolates) or 1b (20 isolates), for which only partial sequences are available in the Los Alamos HCV database. We added these sequences in order to increase the balance of the temporal structure and even dispersion in phylogenetic tree. Otherwise, all of the indications remain the same as that described above for Figure S2. (PPTX) [file pone.0064698.s004.pptx]

## Slide 1
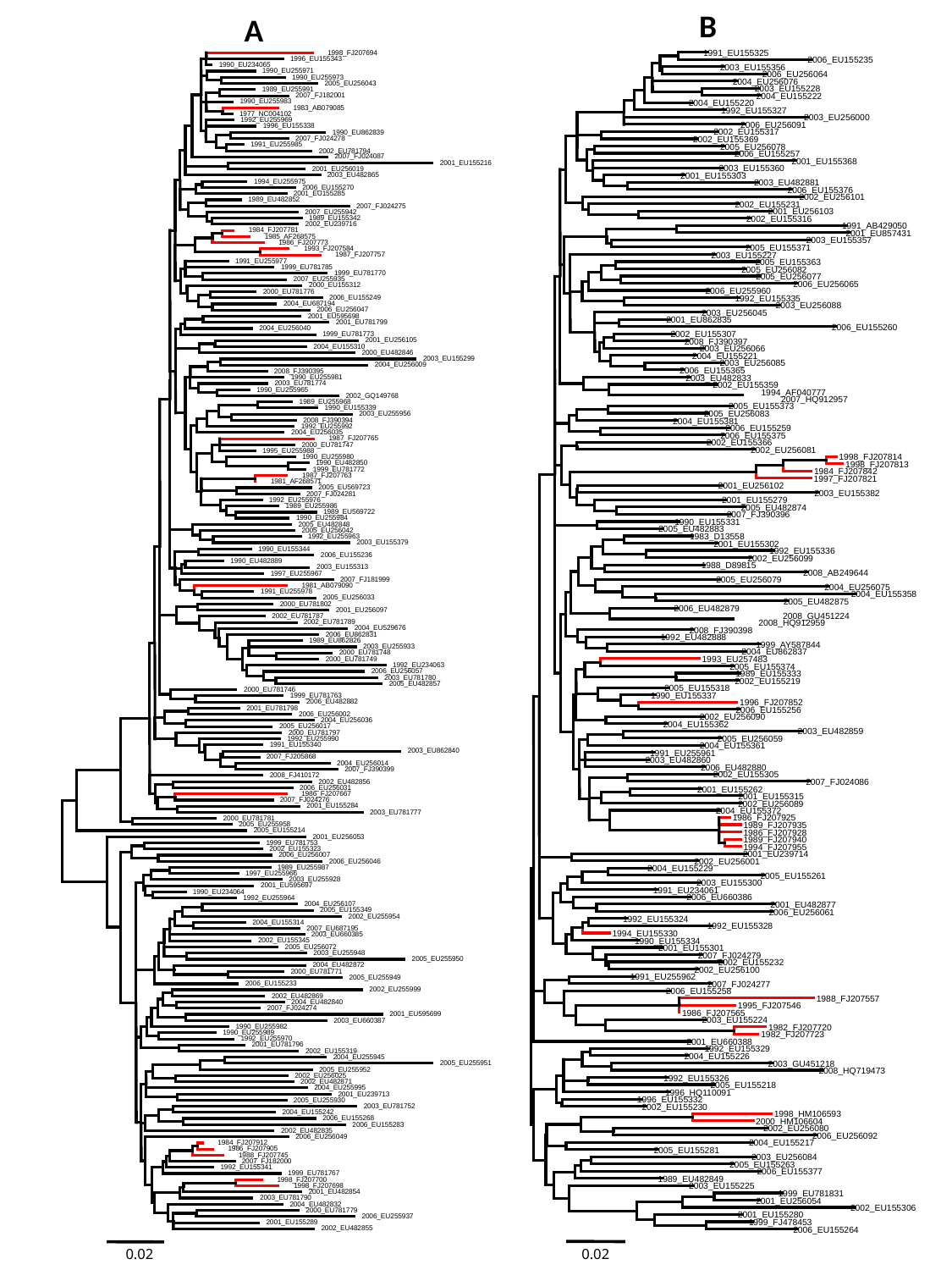

B
A
 1991_EU155325
 2006_EU155235
 2003_EU155356
 2006_EU256064
 2004_EU256076
 2003_EU155228
 2004_EU155222
 2004_EU155220
 1992_EU155327
 2003_EU256000
 2006_EU256091
 2002_EU155317
 2002_EU155369
 2005_EU256078
 2006_EU155257
 2001_EU155368
 2003_EU155360
 2001_EU155303
 2003_EU482881
 2006_EU155376
 2002_EU256101
 2002_EU155231
 2001_EU256103
 2002_EU155316
 1991_AB429050
 2001_EU857431
 2003_EU155357
 2005_EU155371
 2003_EU155227
 2005_EU155363
 2005_EU256082
 2005_EU256077
 2006_EU256065
 2006_EU255960
 1992_EU155335
 2003_EU256088
 2003_EU256045
 2001_EU862835
 2006_EU155260
 2002_EU155307
 2008_FJ390397
 2003_EU256066
 2004_EU155221
 2003_EU256085
 2006_EU155365
 2003_EU482833
 2002_EU155359
 1994_AF040777
 2007_HQ912957
 2005_EU155373
 2005_EU256083
 2004_EU155381
 2006_EU155259
 2006_EU155375
 2002_EU155366
 2002_EU256081
 1998_FJ207814
 1998_FJ207813
 1984_FJ207842
 1997_FJ207821
 2001_EU256102
 2003_EU155382
 2001_EU155279
 2005_EU482874
 2007_FJ390396
 1990_EU155331
 2005_EU482883
 1983_D13558
 2001_EU155302
 1992_EU155336
 2002_EU256099
 1988_D89815
 2008_AB249644
 2005_EU256079
 2004_EU256075
 2004_EU155358
 2005_EU482875
 2006_EU482879
 2008_GU451224
 2008_HQ912959
 2008_FJ390398
 1992_EU482888
 1999_AY587844
 2004_EU862837
 1993_EU257483
 2005_EU155374
 1989_EU155333
 2002_EU155219
 2005_EU155318
 1990_EU155337
 1996_FJ207852
 2006_EU155256
 2002_EU256090
 2004_EU155362
 2003_EU482859
 2005_EU256059
 2004_EU155361
 1991_EU255961
 2003_EU482860
 2006_EU482880
 2002_EU155305
 2007_FJ024086
 2001_EU155262
 2001_EU155315
 2002_EU256089
 2004_EU155372
 1986_FJ207925
 1989_FJ207935
 1986_FJ207928
 1989_FJ207940
 1994_FJ207955
 2001_EU239714
 2002_EU256001
 2004_EU155229
 2005_EU155261
 2003_EU155300
 1991_EU234061
 2006_EU660386
 2001_EU482877
 2006_EU256061
 1992_EU155324
 1992_EU155328
 1994_EU155330
 1990_EU155334
 2001_EU155301
 2007_FJ024279
 2002_EU155232
 2002_EU256100
 1991_EU255962
 2007_FJ024277
 2006_EU155258
 1988_FJ207557
 1995_FJ207546
 1986_FJ207565
 2003_EU155224
 1982_FJ207720
 1982_FJ207723
 2001_EU660388
 1992_EU155329
 2004_EU155226
 2003_GU451218
 2008_HQ719473
 1992_EU155326
 2005_EU155218
 1996_HQ110091
 1996_EU155332
 2002_EU155230
 1998_HM106593
 2000_HM106604
 2002_EU256080
 2006_EU256092
 2004_EU155217
 2005_EU155281
 2003_EU256084
 2005_EU155263
 2006_EU155377
 1989_EU482849
 2003_EU155225
 1999_EU781831
 2001_EU256054
 2002_EU155306
 2001_EU155280
 1999_FJ478453
 2006_EU155264
 1998_FJ207694
 1996_EU155343
 1990_EU234065
 1990_EU255971
 1990_EU255973
 2005_EU256043
 1989_EU255991
 2007_FJ182001
 1990_EU255983
 1983_AB079085
 1977_NC004102
 1992_EU255969
 1996_EU155338
 1990_EU862839
 2007_FJ024278
 1991_EU255985
 2002_EU781794
 2007_FJ024087
 2001_EU155216
 2001_EU256019
 2003_EU482865
 1994_EU255975
 2006_EU155270
 2001_EU155285
 1989_EU482852
 2007_FJ024275
 2007_EU255942
 1989_EU155342
 2002_EU239716
 1984_FJ207781
 1985_AF268575
 1986_FJ207773
 1993_FJ207584
 1987_FJ207757
 1991_EU255977
 1999_EU781785
 1999_EU781770
 2007_EU255935
 2000_EU155312
 2000_EU781776
 2006_EU155249
 2004_EU687194
 2006_EU256047
 2001_EU595698
 2001_EU781799
 2004_EU256040
 1999_EU781773
 2001_EU256105
 2004_EU155310
 2000_EU482846
 2003_EU155299
 2004_EU256009
 2008_FJ390395
 1990_EU255981
 2003_EU781774
 1990_EU255965
 2002_GQ149768
 1989_EU255968
 1990_EU155339
 2003_EU255956
 2008_FJ390394
 1992_EU255992
 2004_EU256035
 1987_FJ207765
 2000_EU781747
 1995_EU255988
 1990_EU255980
 1990_EU482850
 1999_EU781772
 1987_FJ207763
 1981_AF268571
 2005_EU569723
 2007_FJ024281
 1992_EU255976
 1989_EU255986
 1989_EU569722
 1990_EU255984
 2005_EU482848
 2005_EU256042
 1992_EU255963
 2003_EU155379
 1990_EU155344
 2006_EU155236
 1990_EU482889
 2003_EU155313
 1997_EU255967
 2007_FJ181999
 1981_AB079090
 1991_EU255978
 2005_EU256033
 2000_EU781802
 2001_EU256097
 2002_EU781787
 2002_EU781789
 2004_EU529676
 2006_EU862831
 1989_EU862826
 2003_EU255933
 2000_EU781748
 2000_EU781749
 1992_EU234063
 2006_EU256057
 2003_EU781780
 2005_EU482857
 2000_EU781746
 1999_EU781763
 2006_EU482882
 2001_EU781798
 2006_EU256002
 2004_EU256036
 2005_EU256017
 2000_EU781797
 1992_EU255990
 1991_EU155340
 2003_EU862840
 2007_FJ205868
 2004_EU256014
 2007_FJ390399
 2008_FJ410172
 2002_EU482856
 2006_EU256031
 1986_FJ207667
 2007_FJ024276
 2001_EU155284
 2003_EU781777
 2000_EU781781
 2005_EU255958
 2005_EU155214
 2001_EU256053
 1999_EU781753
 2002_EU155323
 2006_EU256007
 2006_EU256046
 1989_EU255987
 1997_EU255966
 2003_EU255928
 2001_EU595697
 1990_EU234064
 1992_EU255964
 2004_EU256107
 2005_EU155349
 2002_EU255954
 2004_EU155314
 2007_EU687195
 2003_EU660385
 2002_EU155345
 2005_EU256072
 2003_EU255948
 2005_EU255950
 2004_EU482872
 2000_EU781771
 2005_EU255949
 2006_EU155233
 2002_EU255999
 2002_EU482869
 2004_EU482840
 2007_FJ024274
 2001_EU595699
 2003_EU660387
 1990_EU255982
 1990_EU255989
 1992_EU255970
 2001_EU781796
 2002_EU155319
 2004_EU255945
 2005_EU255951
 2005_EU255952
 2002_EU256025
 2002_EU482871
 2004_EU255995
 2001_EU239713
 2005_EU255930
 2003_EU781752
 2004_EU155242
 2006_EU155268
 2006_EU155283
 2002_EU482835
 2006_EU256049
 1984_FJ207912
 1986_FJ207905
 1988_FJ207745
 2007_FJ182000
 1992_EU155341
 1999_EU781767
 1998_FJ207700
 1998_FJ207698
 2001_EU482854
 2003_EU781790
 2004_EU482832
 2000_EU781779
 2006_EU255937
 2001_EU155289
 2002_EU482855
0.02
0.02
