# Supplementary material for: The Evolutionary Rates of HCV Estimated with Subtype 1a and 1b Sequences over the ORF Length and in Different Genomic Regions
Source: PLoS One. 2013 Jun 6;8(6):e64698. doi: 10.1371/journal.pone.0064698 (PMC3675120; doi:10.1371/journal.pone.0064698)
Supplement: Table S1 — The related statistics (mean ± Stderr) generated in the Bayesian MCMC analysis of the subtype 1a dataset. (DOCX) [file pone.0064698.s005.docx]

| Table S1. The related statistics (mean ± Stderr) generated in the Bayesian MCMC analysis of the subtype 1a dataset | | | | | | | | | | | | | |
| --- | --- | --- | --- | --- | --- | --- | --- | --- | --- | --- | --- | --- | --- |
|  | **Statistic** | **Core** | **E1** | **E2** | **P7** | **NS2** | **NS3** | **NS4** | **NS5A** | **NS5B** | **Full-ORF** | **Partial C-E1** | **Partial NS5B** |
| **Exponential** | Chain Length (million) | 100 ¶ | 100 ¶ | 300 ¶ | 100 ¶ | 100 ¶ | 100 ¶ | 100 ¶ | 100 ¶ | 300 ¶ | 900 * | 100 ¶ | 100 ¶ |
|  | **Median rate** | **9.04E-04** | **1.45E-03** | **1.92E-03** | **2.15E-03** | **1.33E-03** | **1.09E-03** | **1.29E-03** | **1.23E-03** | **1.03E-03** | **1.56E-03** | **1.56E-05** | **9.77E-04** |
|  | **Median tMRCA** | **46.97** | **71.86** | **79.74** | **75.88** | **85.06** | **75.07** | **68.89** | **92.27** | **81.27** | **89.93** | **56.45** | **77.68** |
|  | Bayesian Factor ‡ | 16.20, 41.46 | 4.82, 25.82 | 2.60, 50.95 | 18.25, 21.46 | 3.43, 21.61 | 2.84, 44.14 | 3.80, 37.76 | 2.97, 45.88 | 3.52, 44.86 | -8.41, 242.08 | 2.60, 31.79 | 27.01, 5.11 |
|  | Posterior | -8415.3±3.3 | -16489.3±3.02 | -33394.37±2.44 | -6536.09±3.77 | -21126.38±5.4 | -45219.47±4.17 | -23084.74±4.384 | -34228.65±3.78 | -35261±5.01 | -232955.29±4.00 | -15177.15±3.35 | -6821.08±3.93 |
|  | treeModel.rootHeight | 51.1±0.56 | 79.6±1.10 | 92.75±3.25 | 90.86±3.79 | 114.37±7.68 | 84.30±2.07 | 81.03±3.43 | 110.68±5.16 | 123.25±24.82 | 105.26±3.72 | 61.77±1.00 | 93.12±1.83 |
|  | Alpha | 0.56±4.1E-3 | 0.74±2.47E-3 | 0.79±9.90E-4 | 0.54±2.37E-3 | 0.80±6.84E-4 | 0.88±5.18E-4 | 0.86±9.8E-4 | 0.69±9.38E-4 | 0.71±3.64E-4 | 0.73±6.13E-5 | 0.71±1.36E-03 | 0.59±1.79E-03 |
|  | pInv | 0.55±1.5E-3 | 0.40±7.3E-4 | 0.41±1.75E-4 | 0.30±9.18E-4 | 0.35±3.24E-4 | 0.51±1.92E-4 | 0.52±3.01E-4 | 0.40±3.47E-4 | 0.53±1.44E-4 | 0.474±2.74E-5 | 0.42±6.20E-04 | 0.50±8.34E-04 |
|  | Uced.mean | 8.9E-4±8.86E-5 | 1.45E-3±1.6E-5 | 1.90E-3±1.73E-5 | 2.16E-3±2.9E-5 | 1.31E-3±2.42E-5 | 1.07E-3±1.82E-5 | 1.27E-3±1.8E-5 | 1.21E-3±1.87E-5 | 1.00E-3±1.60E-5 | 1.53E-3±2.18E-5 | 1.56E-3±2.23E-5 | 9.74E-4±1.25E-5 |
|  | COV | 0.8±6.76E-4 | 0.72±1.3E-3 | 0.688±1.13E-3 | 0.80±7.99E-4 | 0.71±1.71E-3 | 0.69±3.05E-3 | 0.72±1.83E-3 | 0.69±1.77E-3 | 0.68±1.31E-3 | 0.64±1.62E-3 | 0.74±1.02E-03 | 0.796±7.56E-04 |
|  | covariance | -0.019±6.05E-4 | -0.035±6.3E-4 | -2.56E-2±5.20E-4 | -0.02±5.89E-4 | -0.03±6.26E-4 | -0.0045±1.12E-3 | -0.013±7.84E-4 | -0.024±8.04E-4 | -2.27E-2±5.45E-4 | 8.68E-3±9.12E-4 | -0.038±6.15E-04 | -0.023±5.65E-04 |
|  | Treelikelihood | -7277.3±1.3 | -15288.5±1.01 | -32154.05±1.25 | -5305.19±1.29 | -19850.6±1.6 | -43992.56±0.8 | -21917.0±1.1 | -32970.42±3.63 | -34022.59±1.09 | -231663.56±1.68 | -13982.5±1.23 | -5609.73±1.20 |
| **Lognormal** | Chain Length (million) | 100 | 100 | 100 | 100 | 100 | 100 | 100 | 100 | 100 | 100 ¶ | 100 | 100 |
|  | **Median rate** | **8.43E-04** | **1.47E-03** | **1.98E-03** | **2.04E-03** | **1.30E-03** | **1.12E-03** | **1.28E-03** | **1.27E-03** | **1.04E-03** | **1.53E-03** | **1.47E-03** | **9.54E-04** |
|  | **Median tMRCA** | **46.57** | **66.06** | **68.11** | **70.47** | **76.83** | **67.31** | **62.72** | **75.01** | **68.44** | **76.2** | **57.79** | **72.61** |
|  | Posterior | -8430.8±3.358 | -16466.9±1.50 | -33364.8±1.23 | -6519.1±2.95 | -21097.63±2.03 | -45204.24±0.83 | -23078.37±1.55 | -34198.45±1.23 | -35231.4±1.49 | -232920.0±0.79 | -15167.48±1.68 | -6798.44±4.92 |
|  | treeModel.rootHeight | 47.72±0.22 | 66.79±0.11 | 68.51±0.36 | 72.48±0.37 | 78.08±0.22 | 67.7±0.13 | 63.47±0.14 | 75.78±0.15 | 69.12±0.15 | 76.78±0.23 | 58.7±0.15 | 75.28±0.34 |
|  | Alpha | 0.58±5.96E-3 | 0.76±1.87E-3 | 0.80±3.97E-3 | 0.58±4.09E-3 | 0.80±7.06E-4 | 0.89±5.52E-4 | 0.88±1.17E-3 | 0.70±7.41E-4 | 0.71±4.35E-4 | 0.73±01.52E-4 | 0.71±1.26E-03 | 0.61±2.90E-03 |
|  | pInv | 0.55±2.21E-3 | 0.40±5.47E-4 | 0.41±2.73E-4 | 0.31±1.20E-3 | 0.35±3.86E-4 | 0.51±1.84E-4 | 0.52±2.74E-4 | 0.40±3.16E-4 | 0.53±2.36E-4 | 0.47±9.38E-5 | 0.42±5.59E-04 | 0.51±1.25E-03 |
|  | Ucld.mean | 8.47E-4±4.28E-6 | 1.47E-3±3.93E-6 | 1.98E-3±4.68E-6 | 2.05E-3±1.57E-5 | 1.30E-3±5.06E-6 | 1.12E-3±2.32E-6 | 1.28E-3±2.99E-6 | 1.27E-3±2.52E-6 | 1.04E-3±2.24E-6 | 1.53E-3±4.58E-6 | 1.47E-3±3.65E-6 | 9.60E-4±5.03E-6 |
|  | COV | 0.355±9.52E-4 | 0.212±4.88E-4 | 0.23±8.77E-4 | 0.19±2.07E-3 | 0.18±7.27E-4 | 0.18±3.65E-4 | 0.22±4.03E-4 | 0.21±5.24E-4 | 0.21±4.89E-4 | 0.19±2.44E-4 | 0.74±1.02E-03 | 0.1997±1.92E-03 |
|  | covariance | 2.21E-3±6.54E-4 | -4.0E-4±5.94E-4 | -2.0E-4 ±5.69E-4 | -2.3E-3±5.57E-4 | -1.3E-3±5.57E-4 | 3.75E-3±5.78E-4 | 4.49E-3±5.87E-4 | 4.1E-3±5.97E-4 | 3.71E-3±6.10E-4 | 0.01372±6.84E-4 | -0.038±6.15E-04 | -0.003±5.53E-04 |
|  | Treelikelihood | -7307.1±1.8 | -15301.0±1.06 | -32157.0±1.64 | -5339.83±1.58 | -19856.06±1.30 | -43997.96±0.73 | -21923.22±1.40 | -32979.91±0.93 | -34028.42±1.22 | -231654.10±0.63 | -13989.9±1.23 | -5661.37±1.40 |
| **Strict** | Chain Length (million) | 300 | 100 | 300 | 100 | 100 | 300 | 100 | 100 | 100 | 170 | 100 | 100 |
|  | **Median rate** | **7.78E-04** | **1.43E-03** | **1.92E-03** | **1.94E-03** | **1.26E-03** | **1.12E-03** | **1.24E-03** | **1.24E-03** | **9.45E-04** | **1.54E-03** | **1.40E-03** | **9.26E-04** |
|  | **Median tMRCA** | **50.09** | **66.82** | **69.43** | **72.73** | **79.08** | **67.12** | **63.52** | **75.12** | **72.46** | **71.44** | **60.21** | **74.27** |
|  | Posterior | -8521.10±1.06 | -16534.32±1.12 | -33509.41±0.81 | -6544.92±3.22 | -21159.19±0.97 | -45324.33±0.50 | -23187.3±1.22 | -34324.15±0.61 | -35365.0±1.14 | -233512.9±29.00 | -15256.36±1.54 | -6829.15±3.82 |
|  | treeModel.rootHeight | 51.91±0.63 | 67.32±0.10 | 69.70±0.12 | 74.37±0.37 | 79.98±0.16 | 67.36±6.23E-2 | 63.99±0.10 | 75.50±0.11 | 72.860±0.16 | 71.51±5.74E-2 | 60.84±0.12 | 76.78±0.25 |
|  | Alpha | 0.598±3.12E-3 | 0.76±2.1E-3 | 0.80±1.07E-3 | 0.59±2.49E-3 | 0.80±6.2E-4 | 0.89±3.24E-4 | 0.88±7.98E-4 | 0.71±8.04E-4 | 0.72±4.16E-4 | 0.73±1.09E-4 | 0.72±1.27E-03 | 0.61±2.59E-03 |
|  | pInv | 0.554±1.03E-3 | 0.4032±6.95E-4 | 0.412±1.56E-4 | 0.32±8.7E-4 | 0.35±2.9E-3 | 0.51±1.26E-4 | 0.52±2.6E-4 | 0.40±3.28E-4 | 0.53±2.09E-4 | 0.474±6.15E-5 | 0.42±4.58E-04 | 0.51±9.59E-04 |
|  | Clock.rate | 7.83E-4±2.29E-6 | 1.43E-3±2.73E-6 | 1.92E-3±2.53E-6 | 1.95E-3±1.18E-5 | 1.26E-3±2.67E-6 | 1.12E-3±1.13E-6 | 1.24E-4±2.02E-6 | 1.23E-3±1.65E-6 | 9.46E-4±4.95E-6 | 1.54E-3±1.29E-6 | 1.40E-3±2.41E-6 | 9.33E-4±4.43E-6 |
|  | Treelikelihood | -7371.39±0.75 | -15352.79±0.75 | -32276.18±0.73 | -5353.8±1.71 | -19905.6±0.83 | -44098.11±0.45 | -22006.51±0.9 | -33084.65±0.56 | -34133.08±0.78 | -232249.86±28.9 | -14059.97±1.26 | -5684.17±1.15 |
| ¶ Best fitting model based on Bayesian Factor comparison. * Three analyses (300 million + 300 million + 300 million) were performed and the log files were combined. ‡ Log10 Bayesian Factors were calculated and two numbers were shown in each cell. The first number was obtained by comparing with the lognormal model, while the second obtained by comparing with the strict model. Positive vales are favoring the exponential model while negative are favoring the other model that was compared. | | | | | | | | | | | | | |
