# Supplementary material for: The Evolutionary Rates of HCV Estimated with Subtype 1a and 1b Sequences over the ORF Length and in Different Genomic Regions
Source: PLoS One. 2013 Jun 6;8(6):e64698. doi: 10.1371/journal.pone.0064698 (PMC3675120; doi:10.1371/journal.pone.0064698)
Supplement: Table S2 — The related statistics (mean ± Stderr) generated in the Bayesian MCMC analysis of the subtype 1b dataset. (DOCX) [file pone.0064698.s006.docx]

| Table S2. The related statistics (mean ± Stderr) generated in the Bayesian MCMC analysis of the subtype 1b dataset | | | | | | | | | | | | | |
| --- | --- | --- | --- | --- | --- | --- | --- | --- | --- | --- | --- | --- | --- |
|  | **Statistic** | **Core** | **E1** | **E2** | **P7** | **NS2** | **NS3** | **NS4** | **NS5A** | **NS5B** | **Full-ORF** | **Partial C-E1** | **Partial NS5B** |
| **Exponential** | Chain Length (million) | 100 ¶ | 100 ¶ | 400 | 100 ¶ | 100 ¶ | 100 | 100 ¶ | 100 ¶ | 300 | 900 * | 300 ¶ | 200 |
|  | **Media rate** | **5.92E-4** | **1.20E-3** | **1.72E-3** | **1.01E-3** | **1.56E-3** | **9.20E-4** | **1.10E-3** | **9.70E-4** | **4.97E-4** | **1.25E-3** | **1.02E-3** | **4.85E-4** |
|  | **Median tMRCA** | **95.89** | **75.12** | **74.89** | **103.27** | **80.94** | **101.03** | **88.16** | **116.77** | **118.16** | **94.18** | **96.54** | **274.14** |
|  | Bayesian Factor ‡ | 7.28, 30.2 | 1.98, 18.96 | -∞, 34.22 ƚ | 11.52, 6.16 | 1.62, 18.81 | -0.38, 40.53 | 4.04, 10.11 | 1.34, 46.21 | -3557.8, -3519.4 | -2.205, ∞ ƚ | 4.22, 47.99 | -∞, 27.03 ƚ |
|  | Posterior | -7673.11±8.32 | -15205.24±6.08 | -30905.87±4.67 | -6338.15±9.22 | -21590.62±9.56 | -46517.12±10.16 | -23207.01±9.90 | -33559.24±11.07 | -44585.56±7.19 | -230632.46±8.18 | -20678.43±9.79 | -14263.64±22.07 |
|  | treeModel.rootHeight | 288.98±79.1 | 171.78±37.1 | 138.08±15.41 | 390.68±115.56 | 208.19±56.49 | 238.62±46.45 | 364.12±181.16 | 786.53±463.58 | 428.86±101.11 | 900.05±454.80 | 453.05±278.69 | 833.13±5217.15 |
|  | Alpha | 0.53±2.67E-3 | 0.66±1.20E-3 | 0.84±1.58E-3 | 0.89±02.17E-3 | 0.80±8.11E-4 | 0.74±3.85E-4 | 0.79±6.99E-4 | 0.72±4.10E-4 | 0.69±1.3E-3 | 0.71±5.71E-4 | 0.68±1.49E-03 | 0.60±1.48E-3 |
|  | pInv | 0.59±5.72E-4 | 0.40±5.75E-4 | 0.44±1.17E-4 | 0.40±6.22E-4 | 0.37±3.58E-4 | 0.49±2.13E-4 | 0.53±2.99E-4 | 0.40±2.36E-4 | 0.52±6.56E-4 | 0.47±3.42E-5 | 0.41±3.30E-04 | 0.47±7.20E-4 |
|  | Uced.mean | 6.14E-4±1.47E-5 | 1.21E-3±2.86E-5 | 1.73E-3±2.74E-5 | 1.07E-3±2.87E-5 | 1.58E-3±4.88E-5 | 9.51E-4±4.24E-5 | 1.13E-3±3.39E-5 | 9.67E-4±4.58E-5 | 5.13E-4±1.29E-5 | 1.27E-3±4.78E-5 | 1.02E-3±2.03E-5 | 5.75E-4±2.86E-5 |
|  | COV | 7.97±7.81E-4 | 0.73±1.43E-3 | 0.69±9.97E-4 | 0.78±9.22E-4 | 0.70±1.54E-3 | 0.69±2.60E-3 | 0.71±1.71E-3 | 0.71±2.38E-3 | 0.64±1.25E-3 | 0.66±3.15E-3 | 0.77±8.93E-4 | 0.80±7.98E-4 |
|  | Covariance | -0.03±6.79E-4 | -0.031±7.7E-4 | -0.037±5.75E-4 | -0.025±6.75E-4 | -0.047±9.22E-4 | -0.033±1.39E-3 | -0.046±1.06E-3 | -3.43E-2±1.12E-3 | -4.824E-2±7.66E-4 | -1.25E-2±1.54E-3 | -3.90E-3±4.91E-4 | -2.63±5.54E-4 |
|  | Treelikelihood | -6580.56±1.07 | -14131.21±1.71 | -29821.08±3.12 | -5237.97±0.875 | -20481.29±0.77 | -45372.46±1.44 | -22079.98±0.90 | -32381.72±0.90 | -43376.91±3.96 | -229486.45±1.15 | -19003.75±1.21 | -11462.18±1.87 |
| **Lognormal** | Chain Length (million) | 100 | 100 | 100 ¶ | 100 | 100 | 100 ¶ | 100 | 100 | 100 ¶ | 100 ¶ | 100 | 300 ¶ |
|  | **Median rate** | **5.06E-4** | **1.15E-3** | **1.76E-3** | **7.19E-4** | **1.50E-3** | **8.65E-4** | **9.02E-4** | **6.39E-4** | **6.33E-4** | **1.14E-3** | **1.08E-3** | **1.69E-4** |
|  | **Median tMRCA** | **93.69** | **71.42** | **66.72** | **127.96** | **75.27** | **99.09** | **99.30** | **153.07** | **119.35** | **94.90** | **81.93** | **638.11** |
|  | Posterior | -7678.56±5.83 | -15180.96±1.46 | -30868.23±2.39 | -6361.74±4.82 | -21574.05±1.29 | -46504.01±1.17 | -23229.30±2.79 | -33602.63±6.61 | -36370.79±1.79 | -230610.66±1.18 | -20306.68±3.06 | -14628.15±16.54 |
|  | treeModel.rootHeight | 239.54±107.70 | 75.33±0.39 | 68.61±0.25 | 216.74±20.62 | 78.34±0.34 | 104.66±0.61 | 106.31±1.93 | 238.44±17.08 | 138.36±2.52 | 97.33±0.55 | 87.08±0.74 | 2242.80±417.01 |
|  | Alpha | 0.56±1.49E-3 | 0.67±1.33E-3 | 0.85±8.81E-4 | 0.94±2.39E-3 | 0.81±8.78E-4 | 0.75±3.85E-4 | 0.81±8.10E-4 | 0.72±4.43E-4 | 0.68±4.40E-4 | 0.71±1.44E-4 | 0.70±1.44E-3 | 0.61±7.23E-4 |
|  | pInv | 0.59±5.93E-4 | 0.40±5.4E-4 | 0.44±2.30E-4 | 0.41±6.09E-4 | 0.37±3.37E-4 | 0.49±2.13E-4 | 0.53±2.94E-4 | 0.40±2.56E-4 | 0.51±2.47E-4 | 0.47±8.83E-5 | 0.41±5.10E-4 | 0.47±4.48E-4 |
|  | Ucld.mean | 5.11E-4±7.18E-6 | 1.16E-3±5.39E-6 | 1.76E-3±9.44E-6 | 7.43E-4±1.04E-5 | 1.50E-3±6.0E-6 | 8.65E-4±4.23E-6 | 9.0E-4±1.33E-5 | 6.4E-4±2.00E-5 | 6.29E-4±5.02E-6 | 1.13E-3±6.66E-6 | 1.08E-3±7.47E-6 | 2.10E-4±5.38E-6 |
|  | COV | 0.40±1.34E-3 | 0.23±7.15E-4 | 0.21±6.72E-4 | 0.19±1.76E-3 | 0.20±8.65E-4 | 0.19±4.57E-4 | 0.144±7.37E-4 | 0.25±1.06E-3 | 0.20±5.0E-4 | 0.18±2.92E-4 | 0.32±1.06E-3 | 0.30±7.49E-4 |
|  | Covariance | 6.06E-4±6.53E-4 | 3.78E-3±6.72E-4 | -1.9E-3±3.56E-4 | -2.4E-3±6.22E-4 | 2.95E-3±6.58E-4 | 5.57E-3±6.55E-4 | -7.0E-4±6.17E-4 | -3.7E-3±6.55E-4 | -7.1E-3±6.34E-4 | 0.036±8.06E-4 | 6.46E-3±5.45E-4 | 2.37E-3±2.61E-4 |
|  | Treelikelihood | -6596.35±0.77 | -14135.13±1.39 | -29812.98±1.74 | -5261.69±0.76 | -20483.52±0.64 | -45373.33±0.78 | -22088.61±0.53 | -32387.27±0.81 | -35201.73±0.78 | -229484.99±0.27 | -19013.60±1.17 | -11501.39±1.30 |
| **Strict** | Chain Length (million) | 100 | 100 | 100 | 100 | 100 | 100 | 100 | 100 | 100 | 100 | 100 | 100 |
|  | **Median rate** | **4.91E-4** | **1.16E-3** | **1.71E-3** | **7.04E-4** | **1.36E-3** | **8.49E-4** | **9.13E-4** | **8.36E-4** | **6.30E-4** | **1.15E-3** | **1.12E-3** | **1.50E-4** |
|  | **Median tMRCA** | **89.18** | **70.76** | **69.61** | **129.21** | **82.77** | **112.22** | **99.88** | **147.20** | **125.13** | **98.94** | **79.71** | **713.46** |
|  | Posterior | -7737.99±1.90 | -15240.23±0.99 | -30973.78±1.56 | -6392.49±5.57 | -21646.72±0.74 | -46617.22±2.17 | -23260.61±1.83 | -33671.25±1.34 | -36469.60±1.15 | -231048.87±0.48 | -20772.752±1.60 | -14753.08±21.38 |
|  | treeModel.rootHeight | 100.52±0.94 | 72.91±0.23 | 70.97±0.66 | 399.95±126.91 | 85.18±0.25 | 115.63±0.70 | 104.04±1.09 | 153.71±1.17 | 131.78±1.23 | 99.36±0.35 | 81.75±0.61 | 1632.23±188.73 |
|  | Alpha | 0.57±1.48E-3 | 0.68±2.15E-3 | 0.85±1.37E-3 | 0.95±2.14E-3 | 0.82±7.02E-4 | 0.75±3.64E-4 | 0.82±6.77E-4 | 0.72±5.09E-4 | 0.68±3.73E-4 | 0.71±1.32E-4 | 0.70±1.73E-3 | 0.62±9.91E-4 |
|  | pInv | 0.59±5.08E-4 | 0.40±7.24E-4 | 0.44±1.95E-4 | 0.41±6.02E-4 | 0.37±2.94E-4 | 0.49±1.83E-4 | 0.53±2.57E-4 | 0.40±2.18E-4 | 0.51±2.02E-4 | 0.47±8.00E-5 | 0.41±4.81E-4 | 0.47±6.73E-4 |
|  | Clock.rate | 4.97E-4±4.28E-6 | 1.16E-3±3.98E-6 | 1.72E-3±6.30E-6 | 7.14E-4±9.92E-6 | 1.36E-3±3.90E-6 | 8.50E-4±5.52E-6 | 9.12E-4±9.46E-6 | 8.37E-4±5.78E-6 | 6.31E-4±2.13E-6 | 1.15E-3±4.63E-6 | 1.12E-3±6.55E-6 | 1.85E-4±7.74E-6 |
|  | Treelikelihood | -6652.66±0.74 | -14181.30±0.79 | -29900.91±1.07 | -5278.60±1.07 | -20534.05±0.49 | -45471.78±1.14 | -22115.92±0.76 | -32496.93±1.05 | -35295.69±0.55 | -229902.55±0.37 | -19116.63±1.09 | -11565.19±2.16 |

¶ Best fitting model based on Bayesian Factor comparison. * Three analyses (300 million + 300 million + 300 million) were performed and the log files were combined. ‡ Two numbers were shown in each cell. The first number was obtained by comparing with the lognormal model, while the second obtained by comparing with the strict model. Positive values are favoring the exponential model while negative are favoring the other model that was compared. ƚ When the lognormal was compared with the strict model.
